# 

**Published:** 1883-05

**Authors:** 


					﻿Vol,IV.
MAY, 1883.
No. 5.
THE
IntaendcntPraetitiimtr:
A Monthly Journal,
DEVOTED TO
MEDICINE, SURGERY, OBSTETRICS, DENTISTRY, PATHOLOGY
AND POPULAR SCIENCE.
W. c. BARRETT, M. D., D. D. S.,'
EDITOR.
Columbia Publishing Company.
$2.50 Per Annum in Advance. -	- Single Copies, 25 Cents.
Entered at the Post-Office, Buffalo, N. Y., as Second-Class matter.
				

